# Societal risk factors for overweight and obesity in women in Zimbabwe: a cross-sectional study

**DOI:** 10.1186/s12889-020-8215-x

**Published:** 2020-01-28

**Authors:** Nancy T. Mangemba, Miguel San Sebastian

**Affiliations:** Department of Epidemiology and Global Health, Umeå University, SE-901-85 Umeå, Sweden

**Keywords:** Overweight, Obesity, Social, Risk factors, Women, Zimbabwe

## Abstract

**Background:**

Overweight and obesity are well-recognized risk factors for various non-communicable diseases. Evidence shows an increasing burden of overweight and obesity in low and middle-income countries, especially in women. Little is known about the risk factors in Zimbabwe. The aim of this study was to determine the socioeconomic risk factors for overweight and obesity in non-pregnant adult Zimbabwean women.

**Methods:**

A cross-sectional study was conducted using the 2015 Zimbabwe Demographic Health Survey (*n* = 8904) data on the adult female population aged 15 to 49. Body mass index (BMI) was calculated by dividing the body weight by height squared. The socio-economic variables studied were age, marital status, residence, province, religion, education, household wealth index, household size, access to mass media and the use of contraception. Prevalence of overweight (BMI ≥ 25–29.9 kg/m^2^) and obesity (BMI ≥30 kg/m^2^) were determined. Simple and multivariable logistic regressions were then used to ascertain any relationships.

**Results:**

The weighted prevalence of overweight and obesity in adult females was 34.2 and 12.3% respectively. The odds for being overweight and obese were significantly higher with increasing age (Adjusted Odds Ratio (AOR 2.76, 95% CI:2.45–3.11 for overweight and AOR 3.24, 95% CI:2.69–3.90 for obesity) with marriage (AOR 1.58, 95% CI:1.38–1.79 for overweight and AOR 1.54, 95% CI:1.27–1.87 for obesity), high wealth status (AOR 4.01, 95% CI:2.93–5.50 for overweight and AOR 6.97, 95% CI:4.08–11.9 for obesity), and the use of hormonal contraception (AOR 1.24, 95% CI:1.07-1.41 for overweight and AOR 1.35, 95% CI:1.10–1.64 for obesity). Additionally, having higher education increased the odds of being obese (AOR 1.44, 95% CI:1.07–1.96) while being Christian increased the odds for being overweight (AOR 1.13, 95% CI:1.00–1.28).

**Conclusions:**

The prevalence of overweight and obesity among women in Zimbabwe was high. The key social factors associated were older age, being married, being wealthy and the use of hormonal contraception. Having a higher education and being Christian also increased the risk of being obese and overweight respectively. The design of multi-faceted overweight and obesity reduction programs for women that focus on increasing physical activity and strengthening of social support systems are necessary to combat this epidemic.

## Background

Being overweight or obese is a growing pandemic with the worldwide prevalence having trebled in 41 years (1975–2016); currently, 39% of adults are overweight (39% men and 40% women) while 13% are obese (11% men and 15% women) worldwide [1]. Overweight and obesity have been documented as major risks factors for many non-communicable diseases (NCDs) including hypertension, dyslipidaemias, type-2 diabetes mellitus, coronary heart disease, stroke and certain cancers [1–3], many of them occurring prematurely (between ages of 30 and 69); and almost three quarters of the global NCD deaths being present in low and middle-income countries [4, 5]. The World Health Organisation (WHO) classifies the risk factors for NCDs as modifiable behavioural and metabolic risk factors. The modifiable behavioural risk factors include tobacco use, excess salt/sodium intake, harmful alcohol use and insufficient physical activity. The metabolic risk factors are raised blood pressure, overweight and obesity, hyperglycaemia and hyperlipidaemia [6].

Understanding overweight and obesity calls to the study of how they develop. The contributing factors to being overweight and obese may be best evaluated when presented in a socio-ecological model [7] that reflects the interconnections between the six influential factors – individual characteristics, behaviours, household circumstances, community determinants and social and policy factors [7–10].

The burden of overweight and obesity has been rising in low-income countries, particularly in Africa and several studies have documented these trends [11–13]. Amugsi’s study looking at data from 24 African countries over 23 years, showed increasing high levels of overweight and obesity; and two countries stood out -Zimbabwe and Egypt - exceeding 20% for levels of overweight (28 and 36% respectively) and for obesity with prevalences of 13 and 34%, respectively [14]. Neupane’s study that included 32 Sub-Saharan countries at a later time showed similar results, with prevalence ranges of 5.6–27.7% and 1.1–23% for overweight and obesity, respectively. Amugsi’s study only focused on the prevalence and time trends for urban females while Neupane’s included rural women too [15].

Zimbabwe continues to have significantly high prevalence rates relative to its African counterparts and its NCD burden is also on the rise. Little is known however on the possible causes and further research is required [5, 16].

Women tend to be more overweight and obese than males around the world and studies have shown that some NCDs have a predilection for women [17]. For instance, the Nurse’s Health study from 1976 to 2005 showed how women when compared to men, had a higher risk of developing type-2 diabetes mellitus and hypertension for each unit increase in BMI and for each 5-kg increase in weight [17–20]. According to the last Zimbabwe Demographic Health Survey (ZDHS) in 2015, the prevalence for overweight and obesity increased from 25 to 35% for women while the prevalence for men fell from 15 to 13% [16].

Despite all the available data on overweight and obesity in the region and in Zimbabwe, no studies have looked in a comprehensive manner at the factors that may lead to becoming overweight and obese in Zimbabwe. Thus, the aim of this study was to determine the socioeconomic risk factors for overweight and obesity in non-pregnant adult Zimbabwean women.

## Methods

Zimbabwe is a low-income country, in the heart of Southern Africa with a population of 16 million. Its income is largely based on farming, mining and tourism. The health system is largely focused on communicable diseases but the government has not been oblivious to the increases in its NCD burden, and it has embarked on prioritising NCD prevention and treatment as part of its National Health Strategy [16].

This is a cross sectional study which aims to determine the socioeconomic risk factors for overweight and obesity in non-pregnant adult Zimbabwean women. Data from the 2015 Zimbabwe Demographic Health Survey (ZDHS) was used, where a nationally representative sample of households were interviewed. From these estimates of basic demographic and health indicators were derived. The survey was conducted from July to December 2015, in both rural and urban areas in each of the country’s ten provinces. Information on sexual and reproductive health, nutritional status, mortality and morbidity rates and behaviours related to ill health was collected. A two-stage cluster sampling method was used, resulting in the selection of 11,196 households who could participate in the survey. Sampling weights were calculated separately for each sampling stage and for each cluster. Eligible participants were women aged 15–49 who were permanent residents of the selected households or visitors who must have stayed in the household the night before the survey. The questionnaire used focused on the female household members; on their characteristics (age, sex, education, relationship to head of household) and their home (source of water, toilet facilities, ownership of material goods and use of mosquito nets). In addition, it collected information on education level, reproductive and obstetric history, breastfeeding practices, preventive health behaviours, work and spousal characteristics [16].

### Measures

#### Dependent variable

The participants had their height and weight measured by trained technicians and BMI calculated by dividing the weight (in kilograms) by the height in metres squared [16]. A few exclusions were made - pregnant women and visitors leaving a sample of 8904 out of the total of 9955. BMI was then converted into three variables, overweight (BMI ≥25), obesity (BMI ≥ 30) and the normal weight group (BMI ≥15.90 to 24.99).

#### Independent variables

The selection of independent variables from published literature and available data was guided by a socio-ecological model. This model has four categories – individual, household, community and social/policy factors [7, 21–24].

##### Individual factors

Age was grouped into three sets, 15–29 years, 30–44 years and 45–49 years. Education was categorised as primary educated or less, secondary and higher or university education. In Zimbabwe, primary school lasts 7 years and high school 6 years. Employment was excluded as it had 48.4% missing data. Contraceptive use at the time of the study was divided into three categories – none, hormonal and non-hormonal.

##### Household factors

The wealth index was used as generated by DHS, with five categories, from poorest to richest. The wealth index was developed as a composite variable using principal component analysis after assessment of their material belongings. Households were given scores based on the number and kinds of consumer goods they own, including television, bicycle or car, source of drinking water, toilet facilities and flooring materials.

Marital status was recoded into a binary variable with married and unmarried categories.

Family size was recoded into three groups: 1–5, 6–10 and > 11 members.

##### Community factors

Community factors were measured in two ways. Residence was made into a binary category with urban and rural settings; while the 10 geographical provinces were regrouped based on the distribution of the ethnic subgroups to try to capture both determinants. These subgroups were based on the three major ethnic groups, the Shona (Mashonaland), the Manyika (Manicaland) and IsiNdebele (Matebeleland).

Religion was recoded into two groups, Christian and Non-Christian.

##### Social/policy factors

Mass media was a merger of four variables – access to internet, newspaper, radio and television. Once combined, two categories were created, access to at least one of the media options or none.

### Statistical analysis

Out of the 11,196 households, 9955 subjects were included in the Women’s questionnaire, of which 8904 (89.4%) were used after generation of independent and dependent variables and after applying the exclusion criteria. Weighting was applied in all the statistical calculations using the svy command in Stata and the statistical significance set at 0.05. First, analyses on both the prevalence of overweight and obesity were done. Then, bivariable logistic regressions were conducted separately for each predictor variable which yielded the crude odd ratios (ORs). From this, the significant variables were regressed together in a multivariable logistic regression to provide the adjusted odds ratios. Multi-collinearity between the predictors was examined using the Variance Inflation Factor (VIF) but all were lower than 5, which is the generally accepted cut-off value [25].

#### Ethical considerations

The DHS obtained ethical clearance from the Ministry of Health ethical committee before the surveys were conducted. Informed consent was obtained from the respondents (written) prior to the interview. Participation was voluntary and the participants identities and information was kept confidential [16].

## Results

### Description of the study sample

The sample of non-pregnant women aged 15–49 years had a mean age of 28.6 ± 9.3 years. Of these, 34.2% were overweight and 12.3% obese. The highest prevalence of both overweight and obesity were seen in the 30–44-year age group. Over half (57.0%) of the sample was married and 40.6% of these were overweight and almost 15.6% obese. The proportion of overweight and obese increased with levels of education, from 27 to 54% and from 7 to 28% for overweight and obese respectively. The majority of the participants lived in rural areas (61.7% versus 38.7%) and most were living in the Mashonaland regions (areas largely populated by the Shona tribes). As expected, most of the overweight (44.6%) and obese (19.5%) were found in urban areas. The sample had nearly the same proportions of Christians to Non-Christians; and the Christians were more overweight and obese than the non-Christians. Overweight and obesity were directly related to increasing wealth. The poorest were least affected with only 18% being overweight and nearly 4% obese; while on the other end of the wealth index, almost 50% were overweight and 23% obese. Most of the household size was between 1 and 5 (60%). The rate of overweight and obesity fell with increasing household size. Just over three quarters of women had access to mass media (78%) and 47% use hormonal contraception. Women with access to mass media were more overweight and obese than the women without. The overweight and obesity distribution among those using hormonal and non-hormonal contraception was similar (around 40%). For contraceptive users the overweight and obese were more than for non-contraceptive users. Table 1 shows the characteristics of the study sample, also stratified by overweight and obesity.

**Table 1:** Characteristics of study sample and obesity and overweight prevalences in relation to socio-economic risk factors

Variables	Total Sample (%)	Overweight (%)	Obese (%)
Total Sample	8904 (100.00)	3171 (34.17)	1195 (12.33)
Individual Factors			
Age (μ = 28.63 SD 9.3)			
15–29	4892 (54.62)	1156 (22.60)	309 (5.84)
30–44	3442 (39.15)	1709 (47.72)	725 (19.31)
≥ 45	570 (6.23)	306 (50.96)	157 (25.37)
Educational Level			
Primary or less	2225 (27.09)	655 (27.99)	195 (7.84)
Secondary	5946 (65.54)	2105 (34.46)	775 (12.38)
Higher	733 (7.37)	411 (54.34)	221 (28.43)
Contraception			
None	4234 (47.95)	1271 (27.39)	409 (8.61)
Hormonal	4054 (45.85)	1770 (39.94)	665 (15.42)
Non-Hormonal	616 (6.20)	276 (42.86)	117 (18.33)
Household Factors			
Number of Household Members			
1–5	5779 (60.09)	2167 (36.14)	781 (13.10)
6–10	3191 (35.91)	1038 (31.41)	370 (11.25)
> 11	368 (3.99)	112 (27.01)	40 (9.82)
Marital Status			
Married	4982 (57.12)	2143 (40.63)	860 (15.61)
Unmarried	3922 (42.88)	1028 (25.57)	331 (7.96)
Wealth Index			
Poorest	1334 (17.1)	250 (18.98)	47 (3.48)
Poorer	1294 (16.88)	323 (24.62)	82 (6.08)
Middle	1392 (17.59)	431 (30.47)	124 (8.60)
Richer	2279 (22.95)	929 (40.2)	339 (14.82)
Richest	2605 (25.47)	1238 (47.84)	599 (22.76)
Community Factors			
Place of Residence			
Urban	4060 (38.72)	1813 (44.68)	797 (19.48)
Rural	4844 (61.28)	1358 (27.53)	394 (7.82)
Province			
Manicaland	891 (12.43)	313 (31.15)	114 (9.96)
Mashonaland	4679 (60.59)	1692 (35.46)	612 (12.87)
Matebeleland	3334 (26.98)	1166 (32.68)	465 (12.21)
Religion			
Non- Christian	3922 (47.44)	1210 (29.36)	391 (9.06)
Christian	4982 (52.56)	1961 (38.52)	800 (15.28)
Social and Policy Factors			
Access to Mass Media (Television, Newspaper, Radio, Internet)			
Yes	7054 (77.6)	2715 (37.09)	1081 (14.23)
No	1850 (22.4)	2715 (37.09)	110 (5.74)

% are weighted. N(unweighted) = 9955

### Bivariable regression

Each independent variable was run using a weighted logistic regression for both overweight and obesity, and the results are shown in Table 2.

**Table 2:** Risk factors for overweight and obesity. Crude and Adjusted OR with 95% confidence intervals

	Overweight		Obesity	
	Crude OR (95% CI)	Adjusted OR (95% CI)	Crude OR (95% CI)	Adjusted OR (95% CI)
Individual Factors				
Age				
15–29	1	1	1	1
30–44	3.13 (2.80–3.50)	2.76 (2.45–3.11)	3.86 (3.28–4.53)	3.24 (2.69–3.90)
≥ 45	3.57 (2.90–4.38)	3.68 (2.95–4.60)	5.48 (4.34–6.91)	5.74 (4.45–7.43)
Educational Level				
≤ Primary	1	1	1	1
Secondary	1.35 (1.20–1.52)	1.03 (0.91–1.18)	1.66 (1.37–2.00)	1.08 (0.89–1.32)
Higher	3.06 (2.48–3.78)	1.23 (0.95–1.58)	4.67 (3.63–5.99)	1.44 (1.07–1.96)
Contraception Use				
None	1	1	1	1
Hormonal	1.74 (1.55–1.96)	1.24 (1.07–1.41)	1.94 (1.64–2.28)	1.34 (1.10–1.64)
Non-Hormonal	2.01 (1.65–2.44)	1.20 (0.63–1.19)	2.38 (1.81–3.13)	1.31 (0.96–1.78)
Household Factors				
Marital Status				
Unmarried	1	1	1	1
Married	1.99 (1.77–2.23)	1.58 (1.38–1.79)	2.14 (1.81–2.53)	1.54 (1.27–1.87)
Wealth Index				
Poorest	1	1	1	1
Poorer	1.39 (1.14–1.71)	1.40 (1.19–1.74)	1.80 (1.21–2.66)	1.74 (1.16–2.60)
Middle	1.87 (1.56–2.24)	1.82 (1.48–2.23)	2.61 (1.85–3.69)	2.29 (1.58–3.34)
Richer	2.86 (2.38–3.45)	2.91 (2.25–3.74)	4.83 (3.45–6.76)	4.37 (2.82–6.77)
Richest	3.91 (3.29–4.67)	4.01 (2.93–5.50)	8.18 (5.87–11.39)	6.97 (4.08–11.9)
Community Factors				
Place of Residence				
Urban	1	1	1	1
Rural	0.47 (0.41–0.53)	1.01 (0.79–1.29)	0.35 (0.29–0.42)	0.97 (0.68–1.40)
Province				
Manicaland	1	1	1	
Mashonaland	1.21 (1.00–1.47)	0.95 (0.78–1.15)	1.34 (0.99–1.80)	
Matebeleland	1.07 (0.88–1.30)	0.95 (0.77–1.17)	1.28 (0.92–1.72)	
Religion				
Non-Christian	1	1	1	1
Christian	1.51 (1.35–1.68)	1.13 (1.00–1.28)	1.81 (1.57–2.08)	1.12 (0.95–1.33)
Social and Policy Factors				
Access to Mass Media (Television, Newspaper, Radio, Internet)				
No	1	1	1	1
Yes	1.86 (1.62–2.13)	1.12 (0.96–1.31)	2.72 (2.17–3.42)	1.24 (0.96–1.61)
Household				
Number of Household Members				
1–5	1	1	1	1
6–10	0.82 (0.73–0.91)	0.91 (0.80–1.03)	0.84 (0.71–0.99)	0.98 (0.81–1.18)
> 11	0.62 (0.46–0.83)	0.86 (0.63–1.19)	0.72 (0.49–1.06)	1.18 (0.79–1.78)

### Overweight and obesity

The likelihood of being overweight or of being obese increases for older women with a higher education, with access to mass media who use hormonal contraception, are married; are Christian and who live in urban areas regardless of family size. The relationship was stronger for obesity than for overweight.

### Multivariable regression

The statistically significant variables from the bivariable regression analysis were included in a multivariable logistic regression for both outcomes (Table 2). The same variables were statistically significant for both overweight and obesity. With higher education being associated with obesity alone and being Christian with becoming overweight.

After adjusting, the odds for being overweight and obese were significantly higher with increasing age (Adjusted Odds Ratio (AOR 2.76, 95% CI:2.45–3.11 for overweight and AOR 3.24, 95% CI:2.69–3.90 for obesity) with marriage (AOR 1.58, 95% CI:1.38–1.79 for overweight and AOR 1.54, 95% CI:1.27–1.87 for obesity), high wealth status (AOR 4.01, 95% CI:2.93–5.50 for overweight and AOR 6.97, 95% CI:4.08–11.9 for obesity) and the use of hormonal contraception (AOR 1.24, 95% CI:1.07–1.41 for overweight and AOR 1.34, 95% CI:1.10–1.64 for obesity). Additionally, having higher education increased the odds of being obese (AOR 1.44, 95% CI:1.07–1.96) while being Christian increased the odds for being overweight (AOR 1.13, 95% CI:1.00–1.28)

## Discussion

This study examined the socio-economic risk factors of overweight and obesity in Zimbabwean women aged 15–49. The analysis found that 34.2% of women were overweight and 12.3% obese. The multivariable regression analysis revealed that older age, to be married, to have increasing wealth and the use of hormonal contraception were related to being overweight and obesity. Having a higher education was closely linked to being obese while being Christian contributed to being overweight.

The prevalence rates of this study were much larger relative to the ones seen in other countries using similar DHS survey data. In Neupane’s study that looked at women in 32 African countries between 2005 and 2013, the pooled prevalence of overweight was 15.9 and 6.7% for obesity [26]. Amugsi’s study using DHS data but focusing on urban dwelling women in 24 African countries showed similar results with pooled values of 20% for overweight and 10% for obesity [14]. Our study showed much larger rates in urban women of 44.7% for overweight and 19.48% for obese. Though Neupane’s study only focused on three socio-economic variables – place of residence, education and wealth index, the pooled multivariable logistic regressions found that urban women (OR 2.35,CI 2.31–2.40), those with higher education (OR 1.81 CI 1.78–1.85) and the rich (OR 2.45 CI 2.40–2.50) had higher odds of being overweight and obese [26]. These are similar to our findings. However, in our study having a higher education only influenced chances of being obese and living in a rural or urban area were not statistically significant.

The 2015 Ghanaian study on the double burden of malnutrition among women, that also used DHS data, revealed statistically significant odds ratios similar to our study for wealth, marital status and age [27].

The risk factors found in this study were expected since they have been previously reported in the literature. Older women may struggle to lose excess weight due to the hormonal influences in their bodies [28]. Additionally, almost all the African societies are accepting of and even encourage being overweight and obese in older married women. Overweight and obese women are usually found more attractive and seen as healthy and fertile. Once married, a woman is also expected to gain weight so as to show of her husband’s affluence and of marital bliss [29, 30]. A mother is also expected to be an authoritative figure who can command respect from her children, and a larger body size assists in this. This notion of power and authority is then coupled with how the community expects people in positions of authority to be big, fostering the idea that being overweight and obese is acceptable. Puoane et al. did a study on community health workers in South Africa, showing that almost all of them (95%) were overweight and obese, which points to the community’s relation of overweight and obesity with good health, authority and respect [30].

Another issue associated with overweight and obesity particularly in women is the individual perception of current body image [31]. Most women have individual or societal definitions of a desirable body size that are not based on medical measurements. Despite knowing the consequences of overweight and obesity, moderately obese women tend to be found more desirable than normal weight women [30, 31].

In a Kenyan study on slum resident women, they were asked to assess their own current body size and their desired body size using 18 silhouettes ranging from thin to very obese [32]. More than half (53.7%) of the study population who were overweight or obese, underestimated their weight and more than half of women who were classified as overweight or obese indicated a preference for overweight or obese images. Regardless of BMI category, substantial proportions of women indicated a desire to have a body size that was larger than their actual size [32].Zimbabwe, like many other African countries, has also been experiencing rapid changes to its nutritional and lifestyle patterns. There are changes in how much and what is consumed, with increasing refined food, which usually is laden with sugar, fat and salt. Modernisation and globalisation are contributing to more sedentary lifestyles, more automated and mechanised livelihoods with little physical activity. These by-products of urbanisation are mostly available to the wealthy. The economic gap between the rich and poor in Zimbabwe has increased as the country has been experiencing economic decline. The middle class has almost disappeared, the wealthier becoming wealthier and the poor poorer [33]. Being overweight or obese is associated with being wealthy and so there is always a societal pressure to surpass your current wealth level and so there is an underlying force encouraging gaining more weight and keeping it on so as to keep up the appearances of being wealth- resulting in a vicious circle of weight gain ensuing [34]. Richer women, tend to be more educated, have more resources, more knowledge on the importance of healthy eating and physical activity, more understanding of the deleterious effects of overweight and obesity but the socio-cultural barriers most likely prevent them from applying the knowledge and using their wealth access to combat overweight and obesity [27, 34]. This relationship between wealth and overweight and obesity has been shown in most studies done in Africa [14, 27, 34–37].

Our study found an association between the use of hormonal contraception and overweight and obesity. Overall the literature has failed to support this finding. A 2013 meta-analysis recently found no evidence to supporting a causal association [38]. More research is required to support the hormonal contraception – overweight and obesity relationship found in this study.

For several decades, religion has been believed to play a salient role in one’s health. For religiously committed persons, teachings from their faith provide guidance on attaining, maintaining and recovering physical health [39]. A few studies have shown a positive relationship between religion and body weight [23]. Similar to our study, Lapane et al. showed that church members were more likely to be overweight than non-church members [40]. Several reasons have been proffered to explain this relationship [41–43]:

i. religious organisations might be a haven for the overweight and obese trying to escape social stigma ii. many participate in “religious media practice”, where people watch or listen to religious programming while accessing food and beverages; working similarly to how screen media promotes overweight and obesity iii. Many religions strongly discourage smoking, which is an appetite suppressant iv. religious gatherings may also promote greater food consumption -as food is used a celebratory good and members encouraged to cook calorie-laden meals to bring to gatherings.

In our study, we also found an association between higher education and obesity. Higher education in this study was defined as university training and beyond. A systematic review showed a positive association between educational attainment and obesity in low-income countries; while the opposite was true for high income countries [44]. Neupane et al. study in several African countries revealed too a similar pattern to our study [15]. This phenomenon among the highly educated is supported by the nutrition transition theory which suggests that as people rise in their socio-economic status so does their weight. Diets become high in saturated fat, cholesterol, sugar and refined carbohydrates and life more sedentary due to working conditions [45].

### Methodological considerations

This study has several strengths. For the DHS surveys, each country is required to follow standardised procedures to collect the data and to use validated questionnaires which helps to assure both the internal and external validity of the results. However, there are also several limitations to be considered. The data used is cross-sectional, so the establishment of causality is not possible. The female population used in this study may not be representative of the entire adult female population, given that the anthropometric measurements in the DHS were restricted to women who had given birth in the five years preceding the survey. Other important predictors of the outcome variable such as physical activity and total energy intake (nutritional history) were not included as they were not available. There are also possible biases in the study such as the recall and interviewer bias. Participants were asked to give answers to a lot of events that occurred in the past and it is likely that some information might have been forgotten or told incorrectly. Participants might also have given positive answers to please the interviewer, the so-called social desirability bias. The extent of these biases was not possible to determine.

## Conclusions

This study aimed to find the socio-economic factors contributing to overweight and obesity in Zimbabwean women aged between 15 and 49. The prevalence of overweight and obesity in adult females was 34.17% and 12.33%, respectively. The findings further showed that the key contributory factors for overweight and obesity were older age, being married, increasing wealth and the use of hormonal contraception. Additionally, having higher education increased odds of being obese while being Christian increased the odds of being overweight. With the increasing evidence of the rise in NCDs and the indisputable risk that overweight and obesity play in their development, urgent action must be taken to address these two health outcomes. Zimbabwe is currently undergoing stages 3 and 5 of the nutrition transition, making it the optimal time to implement policy changes that target overweight and obesity for lasting sustainable approaches to NCD mitigation. Effective women’s programs focused on health education, physical activity and social support to reduce the socio-cultural barriers that promote overweight and obesity are necessary to combat this epidemic.

## Data Availability

The dataset(s) supporting the conclusions of this article is(are) available in the DHS repository: https://dhsprogram.com/

## Abbreviations

BMI: Body Mass Index
CI: Confidence Interval
NCD: Non-Communicable Diseases
NHANES III: 3RD National Health and Nutrition Examination Survey
OR: Odds Ratio
WHO: World Health Organisation
ZDHS: Zimbabwe Demographic Health Survey
